# Working Excessively and Burnout Among Nurses in the Context of Sick Leaves

**DOI:** 10.3389/fpsyg.2020.00285

**Published:** 2020-02-25

**Authors:** Krystyna Kowalczuk, Elżbieta Krajewska-Kułak, Marek Sobolewski

**Affiliations:** ^1^Department of Integrated Medical Care, Medical University of Białystok, Białystok, Poland; ^2^Faculty of Management, Rzeszow University of Technology, Rzeszow, Poland

**Keywords:** burnout, nurse, working excessively, sick leave, mental health, hospital management

## Abstract

**Introduction:**

Nurses are particularly at risk of being affected by professional burnout because of the unique patient – caregiver relationship, which requires strong emotional involvement.

**Aim:**

In this study, we decided to examine the mutual correlations of working excessively and burnout – two basic occurrences affecting the mental well-being of employees – and their relationship with sick leave.

**Materials and Methods:**

The study was conducted among 460 nurses working in 3 hospitals in Poland. The polish version of Maslach Burnout Inventory and the Working Excessively Questionnaire developed by Paluchowski were used to conduct the survey. All the demographic data and data on sick leaves were obtained from surveys in the form of respondents’ self-reports.

**Results:**

The constructed regression model shows that the tendency to work excessively, as assessed by loss of control over work (LCW), perfectionist work style (PWS), and perceived oppressiveness of the organization (OOP) measures, explains 12.4% of the variation in burnout levels. This confirms that overburden with work can be a factor contributing to the increase in burnout measures. The constructed logistic regression model showed that increase in the level of occupational burnout by 1 point, the chance of nurse having at least three sick leaves per year increases 1.029 times (i.e., by about 2.9%). None of working excessively measures affected the frequency of sick leaves.

**Conclusion:**

(1) Excessive workload increases burnout symptoms, which in turn encourages nurses to take sick leave more frequently. (2) The tendency for nurses to overburden themselves with work may be seen by hospital managers as a positive phenomenon, but, based on this study, it is clear that this can only be done in the short term, whereas in the long term it will be clearly detrimental to the organization of hospitals and the quality of care.

## Introduction

A shortage of nursing personnel severely affects the quality of medical services. This problem has been trending in most countries around the world (Buerhaus et al., 2007; Haddad and Toney-Butler, 2019; Haegdorens et al., 2019). Poland, however, has one of the lowest indicators of practicing nurses per 1000 residents among the OECD countries. This, in 2015, was only 5.2. For comparison, the highest indicator was in Switzerland at 18.0; and in neighboring countries it was: Denmark – 16.7, Germany – 13.3, Sweden – 11.1, Czechia – 8.0, and Lithuania – 7.7. Moreover, the average for 35 OECD countries was 9.0 (OECD, 2017). According to the data from the Polish Supreme Council of Nurses and Midwives report, the same indicator of practicing nurses in 2015 was only 3.9 in the Podlaskie Voivodeship. This was the lowest in the country. This report also points out a decrease in the number of practicing nurses in Poland in recent years and predicts a continuation of this trend in the following years (Supreme Chamber of Nurses and Midwives, 2017).

The successive decrease in the number of professionally active nurses is affected by such issues as: lack of employment in the profession despite obtained qualifications, financially-motivated emigration, and leaving the profession. The scientific literature has identified a number of factors that are the most frequent reasons for nurses leaving the profession. Among them, low wages, too many responsibilities, health problems, psychosocial burden in the workplace, and burnout are usually indicated (Li et al., 2011; Dall’Ora et al., 2015; de Oliveira et al., 2017).

As a professional group, nurses are highly subject to burnout due to the specific patient and caregiver relationship. This relationship requires emotional involvement, where the caregiver must deal with various possible situations, including the patients’ suffering, fear, aggression, or lack of respect for their work (Leiter and Maslach, 2009; Lewandowska and Litwin, 2009; Nayeri et al., 2009).

Burnout was first defined in Freudenberger (1974) as a state characterized by a sense of physical and mental exhaustion, excessive irritability, impatience combined with cynicism, a tendency to isolate oneself, suppressing the emotions, and feelings of chronic boredom. Burnout is the body’s response to long-term overload with obligations, too many responsibilities and difficult tasks, as well as exhausting, monotonous and boring work, and most of all, to chronic work-related stress. Maslach, the author of the Maslach Burnout Inventory (MBI) that we used in our research, defines burnout as “a syndrome of emotional exhaustion, depersonalization, and a reduced sense of personal achievement that can occur in people working with others in a certain way.” Emotional exhaustion is a feeling of emptiness and depletion of strength caused by excessive psychological and emotional requirements made by the job, or the employee himself/herself by having unrealistic requirements toward his/her own abilities. Depersonalization is a sense of heartlessness, detachment and cynicism toward others, and a lowered sensitivity toward other employees. In contrast, a lowered evaluation of personal achievement is a feeling of wasting time and effort at the workplace (Maslach and Leiter, 2005; Maslach et al., 2009, 2012).

Working excessively is defined as an imbalance between work and home, leisure time, and social relationships (Schaufeli et al., 2009a; Molino et al., 2012). The authors of the Working Excessively Questionnaire (WEQ), which we used in our study, define working excessively as a dysfunctionality that affects the entire life. Accordingly, working excessively is not defined by the intensity of work, but that the work is unnecessary. Working excessively occurs in four aspects: a perfectionist work style, loss of control over work, general views about work, and the perceived oppressiveness of the organization. The perfectionist work style is characterized by an excessive passion for order and an exaggerated pursuit of excellence in performing the entrusted responsibilities. General views about work show to what extent the employee agrees with the normative reasons justifying hard work. The perceived oppressiveness of the organization shows to what extent working excessively results from an economic necessity and fear of losing one’s job or acting in accordance with the organization’s culture. Loss of control over work shows the degree of dependence on work (Paluchowski et al., 2014).

Working excessively is often indicated as one of the symptoms of workaholism (Taris et al., 2005; Schaufeli et al., 2008; Van Beek et al., 2012). However, the scientific literature cites many other reasons that could lead to workaholism. Wrzesniewski described three different ways of relating to work: orientation toward activity, where the main expectation of work is money; orientation toward career, where achieving prestige is the main expectation; and vocation, meaning work in the name of aims beyond the individual (Wrzesniewski and Dutton, 2001). Nursing is not perceived as a prestigious profession in Poland, therefore, in the case of nurses, the first and third occur the most often.

Research by the Supreme Council of Nurses and Midwives indicates that nurses in Poland are overworked. This is primarily due to the fact that the minimum employment standards are set based on the registered health services, the number of beds and the type of ward, rather than the actual needs of the hospital. This results in, among others, one nurse only working a night shift and less personnel on the floor on Sundays and holidays, leading to a greater workload due to an increased number of responsibilities per person. Still, nurses often take on extra shifts to work for absent colleagues because they have a sense of mission about their profession that does not allow them to leave patients unattended, while at the same time they take on additional employment because they are underpaid (Wyderka and Niedzielska, 2016; Supreme Chamber of Nurses and Midwives, 2017).

In this study, we decided to examine the mutual correlations of working excessively and burnout – two fundamental occurrences affecting the mental well-being of employees – and their relationship with sick leave. The aim of such a study was to compare the level of measures of excessive work and burnout among nurses grouped by frequency of using sick leave, and, consequently, the effect of burnout and excessive work on nurses’ sick leave. The results of such an examination should be useful to hospital managers and enable them to take measures to reduce overburdening the nursing personnel with professional responsibilities and to prevent the intensification of burnout. Nurses’ burnout and working excessively are known in the scientific literature, but the interplay of these events from the perspective of sick leave had, until now, not been studied in Poland.

## Materials and Methods

The cross-sectional study was conducted from September 28, 2017 to April 30, 2018, in Poland, in the Podlaskie Voivodeship. It included registered nurses working in three inpatient hospitals and in nine departments. Participation in the study was voluntary, and all procedures were approved by the Institutional Review Board of the Medical University of Białystok, ref. no. R-I-002/296/2017.

### Study Group Selection

The selection of respondents to the study group was based on the register of associated nurses in the District Chamber of Nurses and Midwives in Białystok. The total number of registered nurses was 6085 persons (5990 women and 95 men). The selection criterion was employment based on employment contract in a hospital, in the psychiatric, internal medicine, surgical and emergency departments. Nurses working part-time, elsewhere than hospital and on other than employment contract were excluded. The three largest hospitals in the voivodeship employing 10% of the total workforce were selected to conduct the study.

### Study Procedure

The study used was conducted using paper-based questionnaires. The questionnaires were distributed by researchers to the nurses during their work time. All invited hospitals agreed to the participation of their employees in the study, and to it being conducted during working hours. Participation was voluntary. Before the study, each nurse was informed about the anonymity of the conducted research, and about the possibility of withdrawing from the study without stating a reason. They were asked to complete the surveys in their free time within 2 weeks and send the completed questionnaires in a sealed envelope to the investigators’ address. There were 600 questionnaire surveys distributed, out of which 460 correctly completed questionnaires were obtained. Acceptance rate was 77%. There are no known reasons why 140 respondents did not participate in the study. All the demographic data and data on sick leaves were obtained from surveys in the form of respondents’ self-reports. No incentives were used to encourage participation in the study.

### Study Group

The research group consisted of 460 nurses. The vast majority of the respondents were women (92.4%). The mean age for the studied group was 37.4 ± 12.3 years, with a slightly lower median equal to 34 years. The youngest employee was 22 years old and the oldest was 65. Every fourth person surveyed was not more than 26 years old and every fourth person was not less than 48 years old. Over 65% of the respondents had completed higher nursing education, 20% had secondary education with a specialization and 12.2% had secondary education. The majority of the respondents (80% of all respondents) worked as unit nurses, and 10% as surgical nurses.

### Description of the Questionnaire and the Applied Measures

The research tool was the Polish adaptation of the MBI developed by Pasikowski and Sȩk (Maslach and Leiter, 2005; Pasikowski, 2009), and the Working Excessively Questionnaire (WEQ) developed by Paluchowski et al. (2014).

The Maslach Burnout Inventory measures three dimensions of occupational burnout: emotional exhaustion, depersonalization, personal accomplishment and overall score, which is the average of the detailed measures. These measures are standardized by the authors of the questionnaire for a range of 0–100 points (thus, the results can be compared between measures). The higher the values of these measures, the higher the level of occupational burnout. The MBI original and Polish version both have been extensively validated and both have clear scoring guidelines (Pasikowski, 2009).

The Working Excessively Questionnaire (WEQ) used in the study is the latest version of the questionnaire developed by the Paluchowski team between 2004 and 2013. The WEQ has been extensively validated and has clear scoring guidelines (Paluchowski et al., 2014). The questionnaire consists of 65 questions grouped into 4 numerical scores, measuring the signs of excessive workload: Loss of Control over Work (LCW), Perfectionist Work Style (PWS), General Views about Work (GVW), Oppressiveness of the Organization as Perceived (OOP). These measures have not been standardized by the authors of the questionnaire, therefore, their results cannot be compared.

The internal compatibility of the questionnaires was assessed in the examined group of 460 people, with the following Cronbach’s alpha coefficients obtained. For MBI: emotional exhaustion – 0.94, depersonalization – 0.87, lack of work satisfaction – 0.82, general measure – 0.92. For WEQ: loss of control over work – 0.77, perfectionist work style – 0.84, general views about work – 0.87, oppressiveness of the organization – 0.58. The psychometric properties of the questionnaires used are satisfactory – the only exception is the low Cronbach’s alpha value for the last WEQ measure.

### Statistical Methods

Correlations between the measures of working excessively and occupational burnout were analyzed at the level of the entire population using Spearman’s rank correlation coefficient. To examine the occurrence of statistically significant differences in burnout and working excessively measures between groups of nurses with different numbers of sick leaves, the Kruskal–Wallis test was used. The selection of non-parametric methods in these analyzes resulted from the lack of normality in the distribution of MBI and WEQ measures, which was verified using the Shapiro–Wilk test. These correlations were analyzed in pairs (e.g., WEQ with MBI, WEQ with sick leaves, MBI with sick leaves) to receive results that can be compared with other research conducted separately with MBI or WEQ measures. Linear regression analysis was applied to present the combined effect of WEQ components on the overall burnout index. Logistic regression model was constructed to show the impact of MBI and WEQ measures on frequency of sick leaves. Based on the nurse’s responses, they were placed within three groups as shown in Table 1.

**TABLE 1:** Distribution of number of sick leaves.

Sick leaves during the year	Number	Percentage	Mean age (years)
None	218	47.4	35.1
1–2 times	175	38.0	37.5
3 times or more	67	14.6	44.7

## Results

First, the occurrence of occupational burnout and excessive workload in the studied population was analyzed. The average levels of occupational burnout measures range from 32.7 to 38.6% of the maximum value. Herein, it is important to note that 25% of the most burned-out people have a result above 50.7% of the maximum value in the case of the general measure, and even above 66.7% in the case of dissatisfaction with work. The results of job satisfaction loss are the least favorable among the three components of occupational burnout (Table 2).

**TABLE 2:** Occupational burnout measures.

Burnout measures	$\bar{x}$	Me	*S*	*c*_25_	*c*_75_	Minimum	Maximum
Emotional exhaustion	34,8	33,3	30,5	11,1	55,6	0	100
Depersonalization	32,7	20,0	32,0	0,0	60,0	0	100
Lack of work satisfaction	38,6	33,3	30,1	16,7	66,7	0	100
General measure	35,4	32,6	23,6	16,7	50,7	0	100

Next, the levels of measures for excessive workload were analyzed. As in the case of occupational burnout, the results confirm the existence of the excessive workload phenomenon in the study group (Table 3). The biggest burden is the perfectionist work style, where the mean value was 72.3% of the maximum result, and 25% of the most burdened people had a result exceeding 81.1% of the maximum value. The lowest burden turned out to be general views on work, where the average was 48.9% of the maximum result, and 25% of the most burdened people had a result above 57.9% of the maximum value.

**TABLE 3:** Working excessively measures.

WEQ measures	$\bar{x}$	Me	*S*	*c*_25_	*c*_75_	Minimum	Maximum
Loss of control over work LCW	41,3	42	10,1	34	48	16	77
Perfectionist work style PWS	65,1	65	11,0	57	73	28	90
General views about work GVW	46,5	47	12,8	37	55	19	95
Oppressiveness of the organization OOP	33,4	34	6,7	29	38	15	56

Confirmation of occupational burnout and excessive workload presence in the studied population allowed an examination of the correlation between these syndromes. The analysis was performed using the Spearman rank correlation coefficient.

As can be seen from the summary presented in Table 4, the three measures of excessive workload (LCW, GVW, and OOP) are statistically significantly positively correlated with two components of occupational burnout: emotional exhaustion and depersonalization, and with the general measure. Lack of occupational satisfaction shows a statistically significant negative correlation only with the perfectionist work style.

**TABLE 4:** MBI measures vs. WEQ measures.

WEQ measures	MBI measures			
	
	Emotional exhaustion	Depersonalization	Lack of work satisfaction	General measure
Loss of control over work LCW	0.28 (*p* < 0.001***)	0.23 (*p* < 0.001***)	0.08 (*p* = 0.0988)	0.26 (*p* = 0.0000***)
Perfectionist work style PWS	−0.02 (*p* = 0.5989)	−0.03 (*p* = 0.4993)	−0.27 (*p* < 0.001***)	−0.14 (*p* = 0.0031**)
General views about work GVW	0.19 (*p* < 0.001***)	0.15 (*p* = 0.0009***)	−0.04 (*p* = 0.3525)	0.14 (*p* = 0.0025**)
Oppressiveness of the organization OOP	0.27 (*p* < 0.001***)	0.19 (*p* = 0.0001***)	0.09 (*p* = 0.0670)	0.24 (*p* < 0.001***)

** statistically highly significant correlation, *** statistically very highly significant correlation.

Summing up this part of the analysis, it should be mentioned that the correlations between the measures calculated from the MBI and WEQ questionnaires have low statistical strength.

Burnout has an impact on the health of nurses. An analysis of the correlations between measures of MBI and WEQ and the frequency of sick leave use was conducted.

Table 5 summarizes the values of descriptive statistics for MBI measures in terms of being on sick leave. MBI measures (except the lack of work satisfaction) show statistically significant differences between groups of nurses with different levels of absenteeism. The average level of these measures in the first two groups differs from the MBI values in the group of nurses with at least three redundancies per year.

**TABLE 5:** MBI burnout measures and sick leaves.

MBI burout measures	Number of sick leaves during the year									*p*
	
	None (*N* = 218)			1–2 times (*N* = 175)			3 or more (*N* = 67)			
				
	$\bar{x}$	Me	*s*	$\bar{x}$	Me	*s*	$\bar{x}$	Me	*s*	
Emotional exhaustion	27.4	22.2	27.0	36.4	33.3	30.0	54.6	66.7	33.8	<0.001***
Depersonalization	26.8	20.0	29.7	33.5	20.0	30.6	49.9	60.0	36.6	<0.001***
Lack of work satisfaction	36.6	33.3	29.6	37.5	33.3	28.1	48.0	50.0	35.2	0.0584
General measure	30.3	27.0	20.9	35.8	32.2	22.2	50.8	49.6	28.4	<0.001***

*** statistically very highly significant correlation.

Table 6 summarizes the values of descriptive statistics for WEQ measures in terms of being on sick leave. WEQ measures (except PWS) show statistically significant differences between groups of nurses with different levels of absenteeism. The average level of these measures is very similar in the first two groups, which differs from the WEQ values in the group of nurses with at least three redundancies per year.

**TABLE 6:** WEQ working excessively measures and sick leaves.

WEQ	Number of sick leaves during the year									*p*
		
	None (*N* = 218)			1–2 times (*N* = 175)			3 or more (*N* = 67)			
				
	$\bar{x}$	Me	*s*	$\bar{x}$	Me	*S*	$\bar{x}$	Me	*s*	
Loss of control over work (LCW)	40.4	40	9.7	41.4	42	10.5	44.1	45	9.7	0.0116*
Perfectionist work style (PWS)	65.2	66	10.5	65.5	66	11.0	63.2	62	12.2	0.1660
General views about work (GVW)	45.4	45	12.8	46.4	47	12.2	50.5	51	13.5	0.0153*
Oppressiveness of the organization (SOO)	33.0	33	6.7	33.1	34	6.5	35.4	36	6.7	0.0268*

* statistically significant correlation.

Using regression analysis tools, we constructed a model that would enable to describe the combined impact of WEQ components on the overall burnout measure. Herein, the overall burnout rate as a dependent variable and four WEQ components as independent factors were entered into the initial model. Since GVW, as an independent factor, did not have a significant impact on burnout level (*p* = 0.7224), the model parameters underwent re-estimation after the exclusion of this factor. The final version of the model, shown in Table 7, was thus obtained.

**TABLE 7:** Working excessively measures and overall burnout measure.

Working excessively measures (WEQ)	Overall burnout measure		
	
	*R*^2^ = 12.4% *F* = 21.4 *p* = 0.0000***		
	*B*	*p*	β
Loss of control over work (LCW)	0.595	0.0000***	0.254
Perfectionist work style (PWS)	−0.427	0.0000***	−0.198
Oppressiveness of the organization (OOP)	0.466	0.0054**	0.132

** statistically highly significant correlation, *** statistically very highly significant correlation.

The constructed regression model shows that the tendency to work excessively, as assessed by LCW, PWS, and OOP measures, explains 12.4% of the variation in burnout levels. This is not an extremely strong result, yet it does confirm that overburden with work can be a factor contributing to the increase in burnout rates. Accordingly, higher LCW and OOP values correspond to an increase in burnout – 1 LCW point is on average an increase in burnout by 0.595 points, and 1 OOP point is an increase in burnout by 0.466 points. Furthermore, the more a perfectionist work style is followed, the lower the occupational burnout level – an increase in PWS by 1 point translates into a drop in the burnout level by 0.427 points.

The last stage of statistical analysis was to investigate the simultaneous impact of MBI and WEQ measures on the frequency of nurses taking sick leave. The logistic regression model was used. In the constructed model, the number of sick leaves greater than or equal to three times a year relative to the number of sick leaves at the maximum level of 2 was assumed as a dependent variable of dichotomous nature. The independent variables were MBI and WEQ measures and age as a factor affecting the frequency of absenteeism (Table 1). A model with only statistically significant factors was constructed using the progressive step regression procedure. Factors that statistically significantly affect the probability of frequent absenteeism at work are age and the overall measure of occupational burnout. WEQ measures were statistically insignificant and were not used in the final model. The final model is presented in Table 8.

**TABLE 8:** Impact of MBI and WEQ on frequent sick leaves – logistic regression model.

Independent variables	Number of sick leaves during the year (≥3 vs. <3)	
	
	OR (95% c.i.)	*p*
Overall burnout measure [pts]	1.029 (1.017–1.041)	0.0000***
Age [years]	1.051 (1.028–1.075)	0.0000***

OR, odds ratio (with 95% confidence interval). *** statistically very highly significant correlation.

The chance that a nurse will have at least three sick leaves increases with age (by 5.1% each year). An increase in the level of occupational burnout according to the overall MBI measure by 1 point translates into an increase in the chance of frequent absences by 2.9%.

## Discussion

The phenomena of excessive workload and burnout are the subject of many studies, and nurses stand out as one of the most vulnerable occupational groups at risk of burnout. This was found, among others, in studies conducted in Poland where nurses and civil servants were compared (Jaracz et al., 2017) and in Taiwan (Chou et al., 2014), where different groups of healthcare workers were examined.

According to a study conducted on the Polish nursing profession (Znañska-Kozłowska, 2013), age and duration of service do not significantly affect the incidence of occupational burnout. This confirms the need to search for the causes of this phenomenon in other areas. A hypothesis has been made that excessive workload is a factor in the increase of burnout among nurses, because it is recognized that nurses in Poland are overloaded with work. This is evidenced not only by the results of our research, but also by that of the study of Kunecka (2015). This shows that only 6.5% of the examined nurses spend their time in accordance with the accepted standards on breaks at work.

There are many studies available among the various occupational groups showing association between signs of workaholism (Schaufeli et al., 2009b; Jenaabadi et al., 2016, 2017) (a phenomenon in which one of the two pillars is excessive workload) and burnout, usually presented as a general measure. The results of studies conducted among other occupational groups particularly exposed to occupational burnout, such as doctors and teachers, confirm the positive correlation between the components of workaholism and occupational burnout (Taris et al., 2005; Schaufeli et al., 2008). In particular, research conducted among university lecturers in Iran has shown that excessive workload, as part of workaholism, can be a factor predicting the occurrence of each of the three dimensions of burnout defined by Maslach (Hamidizadeh et al., 2014). These results indicate that correlations of these phenomena may also occur in other occupational groups identified as particularly vulnerable to burnout syndrome, including nurses.

The influence of individual manifestations of excessive workload on occupational burnout among nurses has not been comprehensively analyzed so far (Manzano-García and Ayala, 2017). The regression model we developed allowed us to describe the effects of individual components of excessive workload (WEQ) on the overall occupational burnout rate. Based on this, it was concluded that the tendency to overburden oneself with work as indicated by LCW, PWS, OOP measures allows to explain 12.4% of all variability in the level of professional burnout. This is not an extremely “strong” result, but enables a conclusion to be made that being overburdened with work is a predictor of professional burnout. For comparison, the regression model constructed by Spanish researchers explains the variability of the burnout rate only in 4% (Gil-Monte, 2008).

According to our results, higher values of LCW and OOP measures translate into an increase in occupational burnout. In contrast, the more perfectionistic the work style, the lower the professional burnout level. Most studies carried out in this area confirm the existence of certain correlations between these phenomena. A study conducted on a group of Italian nurses by Nonnis has shown that excessive workload affects emotional exhaustion, which is one of the occupational burnout manifestations (Nonnis et al., 2018). Furthermore, the study conducted by Włodarczyk confirmed the negative influence of perfectionism on the formation of occupational burnout (Włodarczyk and Obacz, 2013). Nurses in Iran have also been surveyed, confirming a positive correlation between the overall workload rate and emotional exhaustion, depersonalization, and professional dissatisfaction (Bemana et al., 2013; Asgari et al., 2016). What is more, studies carried out in Italian hospitals have shown the direct effect of workload on burnout, particularly on emotional exhaustion (Portoghese et al., 2014). In addition, researches in Portugal indicate that work overload is one of the predisposing factors of burnout (Queiros et al., 2013). Finally, research conducted in Iran has shown that nurses from departments more exposed to workloads, such as emergency services, are more likely to become victims of occupational burnout (Ahmadi et al., 2014).

Health problems of nurses often originate in psychosocial burdens in the workplace. This is confirmed in numerous scientific studies on the general impact of stress on the physical and mental health of employees as well as in detailed research, e.g., confirming the unequivocally negative impact of bullying on psycho-emotional aspects of nurse health, and indirectly as one of the factors causing burnout on the general condition health of nurses (Arcangeli et al., 2014; Giorgi et al., 2016; Cullati et al., 2017; Nielsen et al., 2019).

The results of our research have shown that occupational burnout among nurses measured by the overall rate is strongly positively correlated with the frequency of taking sick leaves. A similar result was obtained for two components: depersonalization and emotional exhaustion. The lack of correlation with the job satisfaction loss is an exception. Similar results were obtained by researchers in a group of nurses in Greece, where a direct correlation between the physical health of workers and symptoms of burnout was found (Bellali et al., 2007).

Another hypothesis was that occupational burnout and excessive workload increase the probability of the sickness absenteeism of nurses. The subjects of particular interest were nurses, who had to take advantage of the dismissal at least three times a year, because such frequent absences can disrupt the functioning of hospitals. This group constitutes about 15% of the entire population and is about 8–9 years older than nurses with less sickness absence. The logistic regression model we have developed has confirmed the strong effect of general measure of burnout on the frequency of sick leaves taken by nurses. An increase in the overall burnout rate of the MBI questionnaire by 1 point increases the probability that an employee will take at least three sick leaves per year by 2.9%. Similar correlation between burnout and absenteeism was found in the United States (Dyrbye et al., 2019) and Brazil (Da Silva and Diaz Merino, 2017), but no regression model presenting detailed correlation parameters was developed. A Danish team of researchers, however, identified occupational burnout as a predictor of sickness absence among human service workers (Borritz et al., 2010).

The hypotheses made to achieve the aim presented in the introduction have been confirmed by the study results. The negative effect of excessive workload on the burnout syndrome has been confirmed. Thus, increase in professional burnout is conducive to the sickness absenteeism of nurses.

We would like to draw attention to one more obvious correlation. The absence of nurses due to health reasons results in an additional workload for their colleagues remaining in the workplace. Staff shortages mean that nurses replace absent workers, with excessive workloads or additional on-call time. Sometimes they stay in the hospital and fill the shortage of staff in the next shift, immediately after working for one duty shift. They do so either out of inner sense of responsibility that does not allow them to leave patients unattended or they use it as an opportunity to earn extra remuneration. Additional duties naturally contribute to an increase in the workload, which, in turn, has effect on burnout and thus creates a cyclical self-perpetuating mechanism.

## Methodolical Limitations

The sample used, study design (cross-sectional study), lack of adjustment for possible confounders and low Cronbach’s alpha value for “oppressiveness of the organization” measure in WEQ questionaire are significant limitations of the study. The research was conducted only in three hospitals located in a single region of Poland. The financial situation of a country influences the health and workability of workers (Giorgi et al., 2015). Mucci et al. (2016) found that the economic crisis was an important stressor that had a negative impact on workers’ mental health and sickness absence rates. As the economic crisis bypassed Poland, direct comparisons with findings from other studies should be done with caution.

Number of sick leaves were self-reported by the respondents and are influenced by problems that are common to self-report methodology. The reasons for not taking part in the study by 23% of invited persons are unknown due to the manner the study was conducted. It is also unknown whether the nurses who did not participate in the study were more burdened with work than those who participated in the study, and similarly with regard to occupational burnout syndrome.

## Conclusion

(1) Excessive workload and burnout symptoms among nurses interact in such a way that excessive workload increases burnout symptoms, which in turn encourages nurses to take sick leave more frequently. In this way, the resulting sickness absenteeism is a factor that increases the workload for nurses who are obliged to work as replacements for their absent colleagues. Additional duties naturally contribute to an increase in the workload, which in turn has effect on burnout and thus creates a cyclical self-perpetuating mechanism.
(2) The tendency for nurses to overburden themselves with work may be seen by hospital managers as a positive phenomenon, but, based on this study, it is clear that this can only be done in the short term, whereas in the long term it will be clearly detrimental to the organization of hospitals and the quality of care. All the necessary measures must be taken to prevent nurses from becoming excessively overloaded with work.

## Data Availability Statement

The datasets generated for this study are available on request to the corresponding author.

## Ethics Statement

The studies involving human participants were reviewed and approved by the Institutional Review Board of the Medical University of Białystok, ref. no. R-I-002/296/2017. Written informed consent for participation was not required for this study in accordance with the national legislation and the institutional requirements.

## Author Contributions

KK: concept of the research, design of article structure, conducting of the research, review of the literature, results analysis, and writing the article. EK-K: review of the literature and review of article drafts. MS: statistical analysis.

## Conflict of Interest

The authors declare that the research was conducted in the absence of any commercial or financial relationships that could be construed as a potential conflict of interest.
